# Clinical outcomes of carbapenem vs. non-carbapenem therapy in hospitalized adults with shigellosis

**DOI:** 10.1017/ash.2026.10820

**Published:** 2026-08-10

**Authors:** Zach Zhang, Gregory B. Tallman, Brian Kendall, Emily Fox

**Affiliations:** 1 Providence Portland Medical Center, Portland, OR, USA; 2 Providence Saint Vincent Medical Center, Portland, OR, USA

## Abstract

**Background:**

Management of shigellosis is increasingly complicated by antimicrobial resistance, yet susceptibility testing is rarely performed, and the role of broad-spectrum empiric therapy in hospitalized adults remains unclear.

**Methods:**

We conducted a multi-center retrospective cohort study of adults hospitalized with stool-confirmed *Shigella* spp. from October 2023 to August 2024 across five hospitals in the Portland metropolitan area. The primary outcome was time from antibiotic initiation to symptom resolution (less than 3 stools in 24 h) and the secondary outcome was length of hospital stay.

**Results:**

Of 45 patients included in the outcome analyses, 29 (64%) received carbapenems and 16 (36%) received non-carbapenem antibiotics. Median time from antibiotic initiation to symptom resolution was 18 hours (IQR 9–37) in the carbapenem group and 29 hours (IQR 15–36) in the non-carbapenem group, with no significant difference between groups (*P* = .89). The median length of hospital stay was significantly longer in the carbapenem group, at 5.4 days (IQR 3.6–8.3), compared with 3.9 days (IQR 2.6–4.4) in the non-carbapenem group (*P* = .02). Only 26% of patients had susceptibility testing ordered.

**Conclusions:**

Carbapenem use was not associated with faster symptom resolution and was associated with significantly longer hospitalization. Susceptibility testing was underutilized and may be essential to guide local empiric and targeted therapy decisions.

## Background

*Shigella* bacteria are transmitted by the fecal-oral route, either directly through person-to-person contact, or indirectly through contaminated food or water.^1–4^ *Shigella* are easily transmitted, and outbreaks tend to occur among people in close-contact settings. Many cases of shigellosis are self-limiting with supportive care and fluid replacement representing the cornerstone of treatment.^2–4^ Antibiotics may be indicated for patients with severe symptoms to shorten the duration of illness, or to help reduce likelihood of transmission.^3–7^ First-line options have historically included azithromycin, ciprofloxacin, and ceftriaxone.^4^

However, in recent years, multi-drug resistant (MDR) and extensively-drug resistant (XDR) strains of *Shigella* have emerged. An MDR strain is typically defined as an isolate that is resistant to three or more of ampicillin, azithromycin, ciprofloxacin, ceftriaxone, and trimethoprim-sulfamethoxazole (TMP-SMX), and an XDR strain is resistant to all the previously listed antibiotics.^8,9^ The Centers for Disease Control and Prevention published a health advisory alert in 2023, stating approximately 5% of recent *Shigella* infections were caused by XDR strains, up from 0% in 2015.^9^ Unfortunately, clinicians are left with limited treatment options for XDR shigellosis. Antibiotic treatment options that have been proposed include carbapenems or fosfomycin, despite limited clinical outcomes data evaluating effectiveness.^10,11^

There have been multiple outbreaks of *Shigella* across the Portland, Oregon metropolitan area in the past 10 years, primarily among persons experiencing houselessness (PEH).^1,9^ Most recently, there was a significant surge beginning in September 2023.^9^ Local health authorities reported high rates of resistance to first line antibiotics, including fluoroquinolones, macrolides, and third generation cephalosporins. In response, hospitals within our health system began recommending susceptibility testing for all patients with a stool sample positive for *Shigella.* For patients admitted to the hospital with severe symptoms necessitating empiric therapy, we began utilizing short courses (ie, 3–5 d) of carbapenem treatment, consistent with a practice alert issued by our infectious diseases team following consultation with a local county health department during the outbreak. Currently, there is limited clinical data evaluating the effectiveness of carbapenems for the treatment of suspected MDR *Shigella* infections. The purpose of this study was to assess clinical outcomes associated with carbapenem versus non-carbapenem empiric therapy in adults hospitalized with Shigella infection during an outbreak, and to describe local Shigella serotype distributions and resistance testing patterns when this data was available.

## Methodology

### Study design

This was a multi-center retrospective cohort study. Adults (age ≥18 yr) with a stool sample positive for *Shigella* from October 2023 to August 2024 at one of five hospitals within the Portland metropolitan area were eligible for inclusion. Patients were excluded if they had concomitant detection of a gastrointestinal pathogen other than *Shigella*, were started on antibiotics after their documented resolution of symptoms, or if they were seen only in the emergency department (ED) and thus unable to provide longitudinal data. Patients were categorized into three groups based on treatment: carbapenem therapy, non-carbapenem antibiotic therapy, or supportive care alone.

Other data points collected included patient demographic and clinical characteristics such as age, sex, race/ethnicity, housing status, human immunodeficiency virus (HIV) status, immunocompromising conditions, white blood cell (WBC) count, heart rate, respiratory rate, temperature, serum lactate, mean arterial pressure (MAP), and total systemic inflammatory response syndrome (SIRS) criteria met. Immunocompromising conditions were defined as primary or secondary immunodeficiencies, history of solid organ or stem cell transplantation, or receipt of immunosuppressive therapy.^12^ The study was reviewed by the Providence Institutional Review Board (IRB) and deemed exempt from full IRB review.

### Microbiology

*Shigella* spp. was detected from stool using the BD Max Enteric Bacterial Panel (BD Diagnostics, Franklin Lakes, NJ).^13^ If the panel was positive for *Shigella* and viable bacteria could be recovered, specimens were forwarded to the Oregon State Public Health Laboratory for species-level identification and serotyping. Susceptibility testing for ampicillin, ceftriaxone, ciprofloxacin, and TMP-SMX was performed by the designated reference laboratory for clinical microbiology testing across all participating hospitals, when requested by the treatment team. Macrolide sensitivities were not performed. Clinical and Laboratory Standards Institute breakpoints were used for the interpretation of susceptibility results.^14^

### Study outcomes

The primary outcome was time from initiation of antimicrobial treatment to resolution of symptoms. Documented time of symptom resolution was defined as the time the patient experienced less than 3 stools within the subsequent 24-hour period as documented in the electronic medical record. The secondary outcome was the length of hospital stay. Patients who received supportive care alone were included for the purpose of recording serotype and sensitivity data and were excluded from the outcome analyses.

### Statistical analysis

Categorical data were summarized as counts and percentages; continuous data were summarized as medians and interquartile ranges. Baseline characteristics were described descriptively without formal statistical comparisons. The primary outcome of time from antibiotic initiation to symptom resolution was analyzed using Kaplan–Meier curves, and differences between groups were assessed with the log-rank test. The length of hospital stay was analyzed using the Mann-Whitney U test. A *P*-value ≤ .05 was considered statistically significant. Statistical analyses were performed using R software (R Foundation for Statistical Computing, Vienna, Austria).

## Results

A total of 69 patients met initial inclusion criteria. Three patients were excluded due to concomitant detection of another gastrointestinal pathogen, and 16 were excluded as they were only seen in the ED, leaving 50 patients for baseline, serotype, and susceptibility analyses (Tables 1 and 2). Of these, 5 patients received supportive care alone and were excluded from the outcomes analyses, leaving 45 patients in the final outcomes analyses. Among these patients, 29 (64%) patients were treated with carbapenem antibiotics and 16 (36%) were treated with non-carbapenem antibiotics. Baseline patient characteristics are shown in Table 1. Patients who were treated with carbapenems were more likely to be PEH (76%) as well as a person who injects drugs (PWID) (38%). Baseline infectious markers were similar between groups. No patients had septic shock requiring vasopressors. In the non-carbapenem antibiotic group, 7 patients (44%) received ciprofloxacin, 6 patients (38%) received ceftriaxone, 2 patients (13%) received ceftriaxone followed by ciprofloxacin, 1 patient (6%) received azithromycin and ciprofloxacin.

**Table 1.** Baseline characteristics Table 1 long description.

Characteristics	All patients (n = 50)	Carbapenem group (n = 29)	Non-carbapenem antibiotic group (n = 16)	Supportive care group (n = 5)
Age (years)	45 (37–58)	45 (39–58)	45 (30–59)	45 (35–47)
Male	40 (80%)	23 (79%)	12 (75%)	5 (100%)
MSM	7 (14%)	3 (10%)	3 (19%)	1 (20%)
PEH	29 (58%)	22 (76%)	6 (38%)	1 (20%)
HIV	9 (18%)	5 (17%)	3 (19%)	1 (20%)
PWID	12 (24%)	11 (38%)	1 (6%)	0
Gastrointestinal comorbidities	2 (4%)	2 (7%)	0	0
Immunocompromised	2 (4%)	1 (3%)	1 (6%)	0
Acute kidney injury at presentation	24 (48%)	13 (45%)	9 (56%)	2 (40%)
WBC count	11.7 (8.0–16.5)	10.9 (7.5–13.5)	14.0 (11.7–17.4)	10.3 (5.7–19.0)
Number of SIRS criteria^*^ met	2 (1–3)	2 (1–3)	2 (2–3)	2 (2–2)
Serum lactate	1.7 (1.3–2)	1.7 (1.5–2)	1.7 (1.3–1.8)	1.4 (1.3–1.4)
MAP	96 (89–103)	96 (90–101)	96 (89–103)	92 (90–99)
Required vasopressors	0	0	0	0

All data presented as N (%) or median (IQR).

AKI, acute kidney injury; HIV, human immunodeficiency virus; IQR, interquartile range; MAP, mean arterial pressure; MSM, men who have sex with men; PEH, persons experiencing houselessness; PWID, person who injects drugs; SIRS, systemic inflammatory response syndrome; WBC, white blood cell.

* SIRS criteria: temperature >38°C (100.4°F) or <36°C (96.8°F), heart rate >90, respiratory rate >20 or PaCO_2_ <32 mm Hg, WBC > 12,000/mm^3^, <4,000/mm^3^, or >10% bands.

**Table 2.** Microbiology data Table 2 long description.

	All patients (n = 50)	Carbapenem group (n = 29)	Non-carbapenem antibiotic group (n = 16)	Supportive care group (n = 5)
Susceptibility data available	13 (26%)	11 (38%)	2 (103%)	0
Ampicillin susceptible	0/13 (0%)	0	0	0
Ciprofloxacin susceptible	10/13 (77%)	9/11 (82%)	1/2 (50%)	0
Ceftriaxone susceptible	9/10 (90%)	8/9 (89%)	1/1 (100%)	0
Trimethoprim-sulfamethoxazole susceptible	1/11 (9%)	1/9 (11%)	0	0
Species and serotype data available	36 (72%)	18 (62%)	14 (88%)	4 (80%)
*S. flexneri* serotype 1b	25/36 (69%)	15/18 (83%)	9/14 (64%)	1/4 (25%)
*S. flexneri* serotype Y	1/36 (3%)	0	1/14 (7%)	0
*S. flexneri* serotype 2a	2/36 (6%)	1/18 (6%)	0	1/4 (25%)
*S. flexneri* serotype 3a	1/36 (3%)	0	1/14 (7%)	0
*S. sonnei*	7/36 (19%)	2/18 (11%)	3/14 (21%)	2/4 (50%)

All data presented as N (%).

Denominators vary by antibiotic because susceptibility results were not available for every isolate for each agent tested; the specific reason for missing individual results (eg, testing failure, agent not ordered) could not be determined retrospectively.

**Figure 1.**
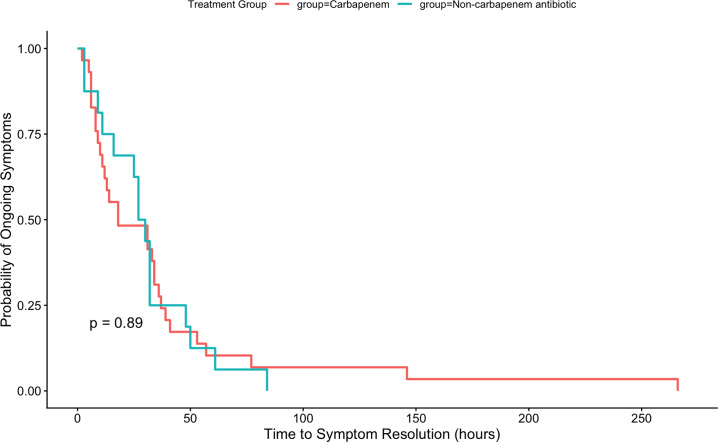
Time from antibiotic initiation to symptom resolution.

The median time from antibiotic initiation to symptom resolution was 18 hours (IQR 9–37) in the carbapenem group and 29 hours (IQR 15–36) in the non-carbapenem group; time to symptom resolution did not differ significantly between groups (*P* = .89) (Figure 1). For the secondary outcome of length of hospital stay, the median length was 5.4 days (IQR 3.6–8.3) in the carbapenem group compared with 3.9 days (IQR 2.6–4.4) in the non-carbapenem group (*P* = .02).

Only 13/50 (26%) cases had susceptibility testing ordered by the treatment team and performed. Susceptibility testing was more common in the carbapenem group, with 11 (38%) isolates undergoing susceptibility testing versus 2 (13%) in the non-carbapenem antibiotic group. Among the 2 cases in the non-carbapenem antibiotic group that had susceptibility data available, both patients received active empiric therapy. Susceptibility results for the commonly tested antibiotics are summarized in Table 2. Ceftriaxone had a susceptibility rate of 90%, followed by ciprofloxacin at 77%. Of the 13 samples that underwent susceptibility testing, 12 were resistant to at least two antibiotic classes tested, and 1 was resistant to all initial antibiotics tested, though upon further testing was susceptible to piperacillin/tazobactam, cefepime, and meropenem.

Most (72%) isolates were sent to the state laboratory for species identification and serotyping. Among typed isolates, 25/36 (69%) were *Shigella flexneri* serotype 1b and 7/36 (19%) were *Shigella sonnei*.

## Discussion

In this multi-center cohort study of adults hospitalized with shigellosis, time from antibiotic initiation to symptom resolution was similar between patients treated with carbapenem and non-carbapenem antibiotics. Notably, most patients received carbapenems rather than non-carbapenem therapy, reflecting adherence to the practice alert issued by our infectious diseases team. The carbapenem group had longer hospitalizations, though notably, time to symptom resolution did not differ significantly between groups, suggesting the discrepancy in length of stay was less likely driven by delayed resolution of shigellosis itself. A higher proportion of patients in the carbapenem group were PEH or PWID, potentially complicating discharge planning. Additionally, carbapenems are only available intravenously, and providers may have been inclined to complete a full treatment course prior to discharge rather than transition to an oral agent. It is also possible that patients selected for carbapenem therapy were more medically complex at baseline, warranting closer monitoring independent of infection severity. Our findings are relevant to antimicrobial stewardship programs, as carbapenem use offered no clear benefit in time to symptom resolution but was associated with longer hospital stays.

Most *Shigella* isolates in our cohort were *S. flexneri*, followed by *S. sonnei.* This contrasts with national surveillance data, where *S. sonnei* typically predominates, reflecting regional differences in circulating strains.^15,16^ *S. flexneri* is more commonly observed among certain adult risk groups, including PEH, MSM, and people with HIV, all of which were well-represented in our study population. Of the 13 isolates for which susceptibility testing was performed, 12 were resistant to at least two antibiotic classes, and one isolate was resistant to all tested agents. Ceftriaxone susceptibility was 90%, which was higher than expected based on previous reports from Oregon and neighboring regions.^15–17^ National data indicate that approximately 5%–30% of *Shigella* isolates demonstrate resistance to ceftriaxone or ciprofloxacin, depending on species and geographic region and year.^15–17^ This variability underscores the challenge of selecting empiric therapy without local susceptibility information. The low proportion of cases that had susceptibility testing ordered in our cohort represents a significant gap in practice. Routine susceptibility testing would allow clinicians to tailor therapy to local epidemiology and minimize unnecessary use of broad-spectrum agents such as carbapenems. Notably, without susceptibility data, clinicians cannot confidently confirm that an oral agent will be effective, which may contribute to prolonged use of intravenous carbapenem therapy and longer hospital stays. Expanding susceptibility testing may represent a modifiable target for reducing unnecessary carbapenem exposure and length of stay.

This study has a few limitations. First, the primary outcome measure is dependent on accurate charting of stool output, which could have been inconsistently recorded. Additionally, stool frequency may not perfectly correlate with infectious cure, and other symptoms such as abdominal cramping or nausea were not captured. However, we selected resolution of diarrhea as the primary outcome because it is more objective and more consistently documented in the medical record in comparison to other symptoms. This is also consistent with previously published shigellosis clinical studies, which have defined therapeutic success based on stool-frequency/consistency and number of watery stools.^6,18^ Second, as is the nature of retrospective studies, selection bias could have occurred, and patients presumed to be more ill at baseline may have been more likely to be treated with carbapenems than non-carbapenem antibiotics. We did attempt to evaluate imbalances between groups by collecting baseline infectious markers, which were mostly similar between carbapenem and non-carbapenem groups. Lastly, as mentioned previously, the relatively low rate of susceptibility testing represents a limitation; however, it also highlights a key gap in clinical practice and supports the need for more routine testing to guide targeted antibiotic therapy and aid in antimicrobial stewardship efforts.

To our knowledge, this is the first study to examine clinical outcomes of empiric carbapenem therapy for shigellosis. In our cohort, carbapenem use was not associated with faster symptom resolution compared with other therapies. Critically, susceptibility testing was performed in only a minority of cases, highlighting a gap in practice that could guide more targeted therapy. Future studies with larger cohorts and more comprehensive susceptibility data are needed to refine empiric treatment strategies and optimize antibiotic use in the contemporary management of shigellosis.

## Table 1. Long description

The table presents baseline characteristics of 50 patients divided into three groups: Carbapenem group (29 patients), Non-carbapenem antibiotic group (16 patients), and Supportive care group (5 patients). The table has 18 rows and 5 columns, including characteristics such as age, gender, MSM, PEH, HIV, PWID, gastrointestinal comorbidities, immunocompromised status, acute kidney injury at presentation, WBC count, number of SIRS criteria met, serum lactate, MAP, and requirement for vasopressors. Each row provides specific data for each group. For example, the age is 45 years for all groups. The male percentage is 80 percent for all patients, 79 percent for the Carbapenem group, 75 percent for the Non-carbapenem antibiotic group, and 100 percent for the Supportive care group. The table also includes various other characteristics and their respective percentages or values across the different groups.

Navigate back to Table 1..

## Table 2. Long description

A table comparing microbiology data across different patient groups. The table has 11 rows and 5 columns. The columns are labeled as All patients (n = 50), Carbapenem group (n = 29), Non-carbapenem antibiotic group (n = 16), and Supportive care group (n = 5). The rows are labeled with various microbiology data points. Row 1: Susceptibility data available, 13 (26 percent), 11 (38 percent), 2 (10 percent), 0. Row 2: Ampicillin susceptible, 0/13 (0 percent), 0, 0, 0. Row 3: Ciprofloxacin susceptible, 10/13 (77 percent), 9/11 (82 percent), 1/2 (50 percent), 0. Row 4: Ceftriaxone susceptible, 9/10 (90 percent), 8/9 (89 percent), 1/1 (100 percent), 0. Row 5: Trimethoprim-sulfamethoxazole susceptible, 1/11 (9 percent), 1/9 (11 percent), 0, 0. Row 6: Species and serotype data available, 36 (72 percent), 18 (62 percent), 14 (88 percent), 4 (80 percent). Row 7: S. flexneri serotype 1b, 25/36 (69 percent), 15/18 (83 percent), 9/14 (64 percent), 1/4 (25 percent). Row 8: S. flexneri serotype Y, 1/36 (3 percent), 0, 1/14 (7 percent), 0. Row 9: S. flexneri serotype 2a, 2/36 (6 percent), 1/18 (6 percent), 0, 1/4 (25 percent). Row 10: S. flexneri serotype 3a, 1/36 (3 percent), 0, 1/14 (7 percent), 0. Row 11: S. sonnei, 7/36 (19 percent), 2/18 (11 percent), 3/14 (21 percent), 2/4 (50 percent).

Navigate back to Table 2..

## References

[1] Hines JZ , Pinsent T , Rees K , et al. Notes from the field: shigellosis outbreak among men who have sex with men and homeless persons - Oregon, 2015–2016. MMWR Morb Mortal Wkly Rep 2016;65:812–813. doi:10.15585/mmwr.mm6531a5. 27513523

[2] Hendrick J , Raqib R , Noor Z , Faruque ASG , Haque R , Petri WA. Shigellosis. Lancet 2025;406:1508–1519. doi:10.1016/S0140-6736(25)01033-5. 40915309 PMC12704457

[3] Christopher PR , David KV , John SM , Sankarapandian V. Antibiotic therapy for Shigella dysentery. Cochrane Database Syst Rev 2010;2010:CD006784. doi:10.1002/14651858.CD006784.pub4. 20091606

[4] CDC. Clinical Care of Shigellosis. Shigella - Shigellosis, 2024. https://www.cdc.gov/shigella/hcp/clinical-care/index.html. Accessed September 25, 2025.

[5] Amoah J , Klein EY , Chiotos K , Cosgrove SE , Tamma PD , for the CDC Prevention Epicenters Program. Administration of a β-lactam prior to vancomycin as the first dose of antibiotic therapy improves survival in patients with bloodstream infections. Clin Infect Dis 2021;ciab865. doi: 10.1093/cid/ciab865. 34606585

[6] Oldfield EC , Bourgeois AL , Omar AK , Pazzaglia GL. Empirical treatment of Shigella dysentery with trimethoprim: five-day course vs. single dose. Am J Trop Med Hyg 1987;37:616–623. doi:10.4269/ajtmh.1987.37.616. 3688315

[7] Kabir I , Butler T , Khanam A. Comparative efficacies of single intravenous doses of ceftriaxone and ampicillin for shigellosis in a placebo-controlled trial. Antimicrob Agents Chemother 1986;29:645–648. doi:10.1128/AAC.29.4.645. 3518625 PMC180459

[8] Magiorakos AP , Srinivasan A , Carey RB , et al. Multidrug-resistant, extensively drug-resistant and pandrug-resistant bacteria: an international expert proposal for interim standard definitions for acquired resistance. Clin Microbiol Infect Off Publ Eur Soc Clin Microbiol Infect Dis 2012;18:268–281. doi:10.1111/j.1469-0691.2011.03570.x. 21793988

[9] Clinician Alert: 2023 drug-resistant Shigella diarrheal illness cluster | Multnomah County. 2023. https://multco.us/news/clinician-alert-2023-drug-resistant-shigella-diarrheal-illness-cluster. Accessed December 8, 2025.

[10] Outbreak of sexually transmitted, extensively drug-resistant Shigella sonnei in the UK, 2021–22: a descriptive epidemiological study - The Lancet Infectious Diseases. https://www.thelancet.com/journals/laninf/article/PIIS1473-3099(22)00370-X/fulltext. Accessed December 8, 2025. 10.1016/S1473-3099(22)00370-X 35809593

[11] Health Alert Network (HAN) - 00486 | Increase in Extensively Drug-Resistant Shigellosis in the United States. 2025. https://www.cdc.gov/han/2023/han00486.html. Accessed December 8, 2025.

[12] Rubin LG , Levin MJ , Ljungman P , et al. 2013 IDSA clinical practice guideline for vaccination of the immunocompromised host. Clin Infect Dis 2014;58:e44–e100. doi:10.1093/cid/cit684. 24311479

[13] BD MAXTM Enteric Bacterial Panel - 442963 | BD. https://www.bd.com/en-us/products-and-solutions/products/product-page.442963. Accessed December 8, 2025.

[14] EM100 Connect - CLSI M100 ED32. 2022. http://em100.edaptivedocs.net/GetDoc.aspx?doc=CLSI%20M100%20ED32:. Accessed January 24, 2023.

[15] Tansarli GS , Long DR , Waalkes A , et al. Genomic reconstruction and directed interventions in a multidrug-resistant shigellosis outbreak in Seattle, Washington: a genomic surveillance study. Lancet Infect Dis 2023;23:740–750. doi:10.1016/S1473-3099(22)00879-9. 36731480 PMC10726761

[16] Libby T , Clogher P , Wilson E , et al. Disparities in shigellosis incidence by census tract poverty, crowding, and race/ethnicity in the United States, foodNet, 2004–2014. Open Forum Infect Dis 2020;7:ofaa030. doi:10.1093/ofid/ofaa030. 32099844 PMC7032626

[17] BEAM Dashboard | CDC. https://www.cdc.gov/ncezid/dfwed/BEAM-dashboard.html. Accessed December 8, 2025.

[18] Martin JM , Pitetti R , Maffei F , Tritt J , Smail K , Wald ER. Treatment of shigellosis with cefixime: two days vs. five days. Pediatr Infect Dis J 2000;19:522–526. doi:10.1097/00006454-200006000-00006. 10877166

